# Comparison of PD-1 Inhibitors in Patients With Advanced Esophageal Squamous Cell Carcinoma in the Second-Line Setting

**DOI:** 10.3389/fonc.2021.698732

**Published:** 2021-09-21

**Authors:** Yi-Xin Zhou, Ping Chen, Yu-Ting Sun, Bei Zhang, Miao-Zhen Qiu

**Affiliations:** ^1^Department of VIP Region, State Key Laboratory of Oncology in South China, Collaborative Innovation Center for Cancer Medicine, Sun Yat-Sen University Cancer Center, Guangzhou, China; ^2^Department of Medical Oncology, State Key Laboratory of Oncology in South China, Collaborative Innovation Center for Cancer Medicine, Sun Yat-Sen University Cancer Center, Guangzhou, China

**Keywords:** esophageal squamous cell carcinoma, PD-1 inhibitor, second line therapy, camrelizumab, nivolumab, pembrolizumab

## Abstract

**Background:**

KEYNOTE-181, ATTRACTION-3, and ESCORT trials have opened the era of programmed death 1 (PD-1) inhibitors in the second-line therapy for esophageal squamous cell carcinoma (ESCC). There is no head-to-head comparison of pembrolizumab *vs.* nivolumab *vs.* camrelizumab in the second-line setting for ESCC. We performed an indirect comparison to explore the optimal choice of immune checkpoint inhibitor (ICI) for advanced ESCC.

**Methods:**

Patients in ATTRACTION-3 and ESCORT were all squamous carcinoma, while KEYNOTE-181 enrolled both adenocarcinoma and squamous carcinoma patients. We only extract information of patients with squamous carcinoma from KEYNOTE 181 study and all the patients from ATTRACTION-3 and ESCORT. The main clinical outcomes for this study were overall survival (OS), progression-free survival (PFS), objective response rate (ORR), and treatment-related adverse events (TRAEs).

**Results:**

Indirect analysis showed similar survival benefit among three PD-1 inhibitors. Nivolumab was comparable with pembrolizumab in most subgroups except that nivolumab was slightly better for patients with performance status (PS) score of 1 [HRnivo/pembro: 0.68 (95% confidence interval (CI): 0.45–1.02], *p* = 0.07). Compared with nivolumab indirectly, pembrolizumab and camrelizumab had better PFS [HRpembro/nivo: 0.85 (95% CI: 0.63–1.14), *p* = 0.29; HRcam/nivo: 0.64 (95% CI: 0.47–0.87), *p* = 0.004] and significantly higher ORR [RRpembro/nivo: 2.51 (95% CI: 1.22–5.15), *p* = 0.01; RRcam/nivo: 3.52 (95% CI: 1.73–7.18), *p* = 0.001]. Compared with camrelizumab indirectly, pembrolizumab had slightly worse PFS [HRpembro/cam: 1.33 (95% CI: 0.99–1.79), *p* = 0.057] and comparable ORR [RRpembro/cam: 0.71 (95% CI: 0.32–1.60; *p* = 0.41)]. Camrelizumab had a significantly higher rate of all grade TRAEs than both pembrolizumab and nivolumab.

**Conclusions:**

Combining the safety and potential survival benefit, we recommend nivolumab for ESCC patients with PS score of 1 and pembrolizumab or camrelizumab for patients with better PS and seeking for higher efficacy or longer PFS.

## Introduction

Metastatic esophageal cancer has poor prognosis, with a 5-year overall survival rate of ≤8% (1). Esophageal squamous cell carcinoma (ESCC) is the dominant histological subtype for esophageal cancer (2). Current chemotherapy options for second-line ESCC including taxol or irinotecan, offer poor survival and are associated with toxicity (3). There is an urgent need for new effective therapy for patients with ESCC.

Programmed death 1 (PD-1) inhibitors showed promising antitumor activity in patients with advanced esophageal carcinoma who were refractory to or intolerant of standard chemotherapies (4–6). Recently, the results of three randomized phase 3 studies in second-line setting for esophageal carcinoma were released. Compared with chemotherapy, pembrolizumab improved survival in PD-L1 combined positive score (CPS) ≥10 subgroups in KEYNOTE-181 study (7). Meanwhile, ATTRACTION-3, a randomized phase III study, showed a statistically significant and clinically meaningful improvement in overall survival (OS) of nivolumab *versus* chemotherapy in patients with advanced ESCC (8). ESCORT study found camrelizumab significantly improved OS in patients with advanced or metastatic ESCC compared with chemotherapy (9).

However, there is no head-to-head comparison of pembrolizumab *vs.* nivolumab *vs.* camrelizumab in the second-line setting for ESCC. Therefore, we performed an indirect comparison of KEYNOTE-181, ATTRACTION-3, and ESCORT studies to explore the optimal choice of anti-PD1 treatment for previously treated advanced ESCC. Furthermore, subgroup-immune checkpoint inhibitors (ICI) interaction test was performed in order to identify subsets of patients who would benefit most in terms of survival from different PD-1 inhibitors.

## Methods

Patients in ATTRACTION-3 and ESCORT were all squamous carcinoma, while KEYNOTE-181 enrolled both adenocarcinoma and squamous carcinoma patients. In the present study, we only extract information of patients with squamous carcinoma from KEYNOTE 181 study.

The main clinical outcomes for this study were OS, progression-free survival (PFS), objective response rate (ORR), and treatment-related adverse events (TRAEs). Hazard ratio (HR) and its 95% confidence interval (CI) were extracted for OS and PFS, while risk ratio (RR) was extracted for ORR and TRAEs. Data were retrieved from the main outcomes of the three clinical trials by two independent investigators (YZ and PC).

We performed indirect analyses to compare the three PD-1 inhibitors using the frequentist methods (10), which indirectly compared arm A *versus* arm B (pembrolizumab *vs.* nivolumab, pembrolizumab *vs.* camrelizumab, and camrelizumab *vs.* nivolumab, respectively), linked by arm chemotherapy (C) with the formula: log HRAB = log HRAC-log HRBC, and $\text{SE}(\log\text{HRAB})=\sqrt{\text{SE}{\left(\log\text{HRAC}\right)}^{2}+\text{SE}{\left(\log\text{HRBC}\right)}^{2}}$ [standard error (SE)]. The same formula was used for RR.

Moreover, subgroup meta-analyses were applied to investigate the subgroup-ICI interaction for OS (11). Five subgroups were available for the analyses, including gender (male *vs.* female), CPS of PD-L1 expression (≥10% *vs.* <10%), performance status (PS) (0 *vs.* 1), region (Asia *vs.* extra-Asia), and age (<65 *vs.* ≥65 years old). In each subgroup of each study, subgroup-ICI interaction was calculated using the indirect analysis mentioned above. We then conducted direct analyses (inverse-variance-weighted method) to calculate the pooled estimates with subgroup data from the three studies. Either fixed-effect or random-effect model was applied according to the heterogeneity.

The statistical analyses were performed using Stata (version 15.0, Stata Corporation, College Station, TX, USA), with a statistical significance setting at *P* <.05 (two-sided).

## Results

A total of 1,178 ESCC patients (329 patients from ATTRACTION-3, 401 patients from KEYNOTE-181, and 448 patients from ESCORT study) were enrolled in the analyses for OS, PFS, and ORR, and 1,475 patients were included in the analysis for TRAEs. **Table 1** presents the main features and outcomes of these three studies.

**Table 1:** Principal features and outcomes in included trials comparing PD-1 inhibitor with chemotherapy.

Source	Histology	Therapeutic regimen	Chemotherapy drug	No. of patients		NO. of response		PFS^a^	HR for PFS	OS^a^ (m)	HR for OS	Median Follow-up time (m)
				PD-1 inhibitor	Chemo	PD-1 inhibitor	Chemo	(m)				
KEYNOTE-181 2020	squamous and nonsquamous^b^	Pembro *vs.* Chemo	1) paclitaxel 2) docetaxel 3) irinotecan	198	203	33	15	2.2 *vs.* 3.1	0.92 (0.75-1.13)	8.2 *vs.* 7.1	0.77 (0.63-0.96)	7.1 *vs.* 6.9
ATTRACTION-3 2019	squamous	Nivo *vs.* Chemo	1) paclitaxel 2) docetaxel	171	158	33	34	1.7 *vs.* 3.4	1.08 (0.87-1.34)	10.9 *vs.* 8.4	0.77 (0.62–0.96)	10.5 *vs.* 8.0
ESCORT 2020	squamous	Cam *vs.* Chemo	1) docetaxel 2) irinotecan	228	220	46	14	1.9 *vs.*1.9	0.69 (0.56-0.86)	8.3 *vs.* 6.2	0.71 (0.57–0.87)	8.3 *vs.* 6.2

^a^Data presented as “PD-1 inhibitor vs. Chemo”.

^b^Only data for squamous cancer were extracted and analyzed.

Pembro, Pembrolizumab; Nivo, Nivolumab; Cam, Camrelizumab; Chemo, Chemotherapy; PD-1, programmed cell death 1; HR, Hazard Ratio; PFS, Progression-free Survival; OS, Overall survival.

**Figure 1:**
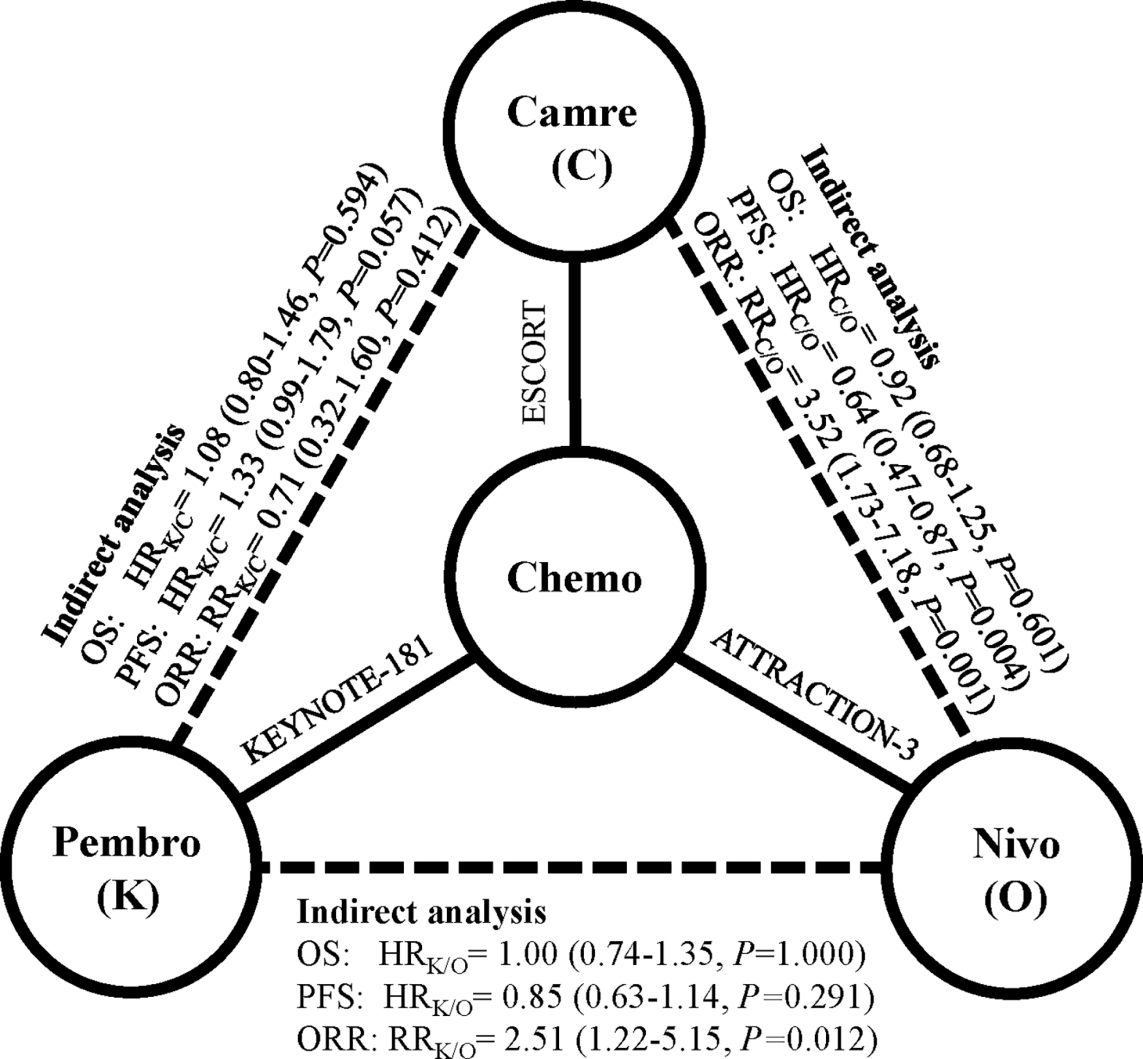
Indirect comparison among pembrolizumab, nivolumab, and camrelizumab in terms of overall survival (OS), progression-free survival (PFS), and objective response rate (ORR). Solid lines represent the existence of direct comparisons between treatment regimens, and dashed line represents the indirect comparison among the three PD-1 inhibitors. All statistical tests were two sided. Abbreviations: Pembro, pembrolizumab; Nivo, nivolumab; Cam, camrelizumab; PD-1, programmed cell death 1; HR, hazard ratio; RR, risk ratio.

Indirect analysis showed similar survival benefit among these three PD-1 inhibitors: HR_pembro/nivo_: 1.00 (95% CI: 0.74–1.35), *p* = 1.000; HR_pembro/cam_: 1.08 (95% CI: 0.80–1.46), *p* = 0.594; H_can/nivo_: 0.92 (95% CI: 0.68–1.25), *p* = 0.601 (**Figure 1**). In subgroup survival analysis compared with chemotherapy, nivolumab improved survival in patients who were younger [HR: 0.65 (95% CI: 0.47–0.89)], male [HR: 0.79 (95% CI: 0.63–0.99)], PS score of 1 [HR: 0.61 (95% CI: 0.45–0.82)], and Asian [HR: 0.78 (95% CI: 0.62–0.97)]; pembrolizumab improved survival in those with PS score of 0 [HR: 0.64 (95% CI: 0.45–0.91)], PD-L1 CPS ≥10 [HR: 0.63 (95% CI: 0.46–0.90)], male [HR: 0.78 (95% CI: 0.63–0.95)], and Asian [HR: 0.63 (95% CI: 0.50–0.80)]; and camrelizumab improved survival in patients with PS score of 1, PD-L1 CPS <10. Indirect analysis showed that these three PD-1 inhibitors were comparable in most subgroups except that nivolumab was slightly better than pembrolizumab for patients with PS score of 1 [HR_nivo/pembro_: 0.68 (95% CI: 0.45–1.02), *p* = 0.07] (**Table 2**).

**Table 2:** Indirect comparisons among pembrolizumab, nivolumab, and camrelizumab in terms of predefined subgroup of OS and treatment-related adverse events.

Subgroup of OS	Pembro/Cam	*P*	Cam/Nivo	*P*	Pembro/Nivo	*P*
**Age, years**						
<65	1.08 (0.75-1.57)	0.68	1.15 (0.77-1.72)	0.48	1.25 (0.82-1.91)	0.31
≥65	1.27 (0.74-2.16)	0.39	0.70 (0.41-1.18)	0.18	0.88 (0.56-1.38)	0.59
**Gender**						
Male	1.04 (0.76-1.43)	0.81	0.95 (0.69-1.30)	0.75	0.99 (0.72-1.36)	0.93
Female	1.78 (0.71-4.43)	0.22	0.63 (0.23-1.66)	0.35	1.11 (0.49-2.54)	0.80
**Performance status**						
0	0.69 (0.37-1.27)	0.23	1.03 (0.57-1.87)	0.91	0.71 (0.44-1.14)	0.15
1	1.33 (0.93-1.89)	0.12	1.10 (0.75-1.60)	0.63	1.46 (0.98-2.18)	**0.07**
**PD-L1 expression**						
≥10	1.07 (0.53-2.13)	0.86	0.87 (0.42-1.81)	0.71	0.93 (0.55-1.57)	0.78
<10	1.24 (0.86-1.77)	0.25	0.89 (0.63-1.25)	0.49	1.10 (0.75-1.61)	0.63
**Region**						
Asia	0.92 (0.64-1.30)	0.63	0.91 (0.67-1.24)	0.55	0.83 (0.58-1.20)	0.32
Extra-Asia	NA	NA	NA	NA	1.81 (0.56-5.89)	0.32
**TRAEs**						
All events	0.71 (0.63-0.79)	**<0.01**	1.52 (1.35-1.71)	**<0.01**	1.08 (0.94-1.24)	0.30
Grade≥3	0.9 (0.60-1.37)	0.63	1.72 (1.11-2.66)	**0.01**	1.55 (1.03-2.34)	**0.03**
Serious events	NA	NA	1.60 (0.88-2.89)	0.12	NA	NA
Leading to discontinuation	0.73 (0.28-1.89)	0.51	1.36 (0.53-3.53)	0.52	0.99 (0.42-2.37)	0.99
Leading to death	0.42 (0.07-2.56)	0.34	3.40 (0.37-31.48)	0.28	1.41 (0.16-12.28)	0.75

Pembro, Pembrolizumab; Nivo, Nivolumab; Cam, Camrelizumab; PD-1, programmed cell death 1; OS, Overall survival; TRAEs, treatment-related adverse events; NA, not applicable.

Bold value means statistically significant or has the trend of significance.

Nivolumab had no clear PFS or ORR benefit towards chemotherapy [PFS—HR_nivo/chemo_: 1.08 (95% CI: 0.87–1.34); ORR—RR_nivo/chemo_: 0.90 (95% CI: 0.58–1.37)], while pembrolizumab had a significantly higher ORR than chemotherapy [RR_pembro/chemo_: 2.26 (95% CI: 1.27–4.02)]. Camrelizumab had comparable PFS and higher ORR than chemotherapy [PFS—HR_cam/chemo_: 0.69 (95% CI: 0.56–0.86); ORR—RR_cam/chemo_: 3.17 (95% CI: 1.80–5.60)] (**Table 1**). Compared with nivolumab indirectly, pembrolizumab had numerically better PFS [HR_pembro/nivo_: 0.85 (95% CI: 0.63–1.14), *p* = 0.29] and significantly higher ORR [RR_pembro/nivo_: 2.51 (95% CI: 1.22–5.15); *p* = 0.01]. Compared with camrelizumab indirectly, pembrolizumab had slightly worse PFS [HR_pembro/cam_: 1.33 (95% CI: 0.99–1.79), *p* = 0.057] and comparable ORR [RR_pembro/cam_: 0.71 (95% CI: 0.32–1.60); *p* = 0.41]. Compared with nivolumab indirectly, camrelizumab had significantly better PFS [HR_cam/nivo_: 0.64 (95% CI: 0.47–0.87), *p* = 0.004] and higher ORR [RR_pembro/nivo_: 3.52 (95% CI: 1.73–7.18), *p* = 0.001].

Analyses of TRAEs suggested that nivolumab was associated with significantly lower rate of ≥ grade 3 TRAEs [RR_pembro/nivo_: 1.55 (95% CI: 1.03–2.34), *p* = 0.03; RR_cam/nivo_: 1.72 (95% CI: 1.11–2.66), *p* = 0.01]. Camrelizumab had a significantly higher rate of all grade TRAEs than both pembrolizumab and nivolumab [RR_pembro/cam_: 0.71 (95% CI: 0.63–0.79), *p* < 0.01; RR_cam/nivo_: 1.52 (95% CI: 1.35–1.71), *p* < 0.01] (**Table 2**). The rates of serious TRAEs and TRAEs leading to discontinuation or death were similar among these three PD-1 inhibitors.

**Figure 2:**
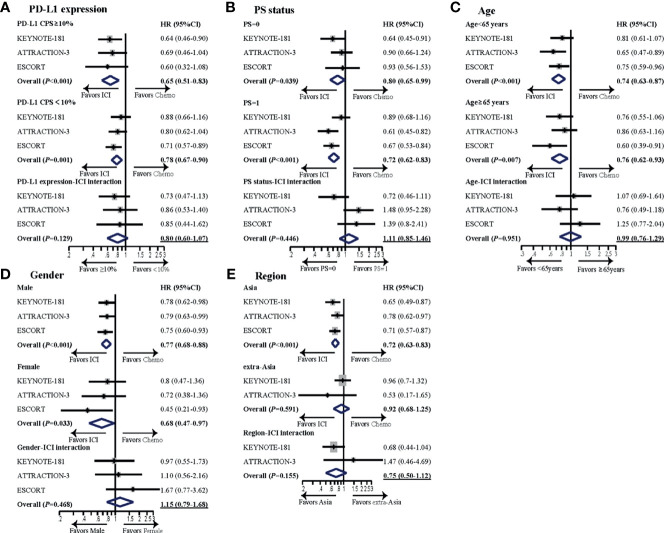
Subgroup meta-analyses investigating survival benefit from PD-1 inhibitors in different subsets of patients. Subgroups analyzed included **(A)** PD-L1 expression, **(B)** PS status, **(C)** age, **(D)** gender, and **(E)** region. For each subgroup, forest plot of hazard ratio (HR) was performed for OS. The horizontal line crossing the dot represents the 95% confidence interval (CI). The dot represented the estimated overall effect, based on the analysis. In each study, subgroup-ICI interaction was calculated using the indirect analysis. Then, meta-analyses were performed for the pooled estimates with all the enrolled studies. The final pooled results of subgroup-ICI interaction were shown by the data with underline. All statistical tests were two sided. PD-L1, programmed cell death ligand-1; CPS, combined positive score; HR, hazard ratio; ICI, immune checkpoint inhibitor; Chemo, chemotherapy; PS, performance status.

In the subgroup analyses (**Figure 2**), no significant difference was found in subgroups including PD-L1 status, PS, age, gender, or region, but PD-L1 expression and region demonstrated a numerical difference of interaction with ICI in terms of OS. Overall HR for ICI *vs.* chemotherapy (reference) was 0.65 (95% CI: 0.51–0.83, *p* < 0.001) and 0.78 (95% CI: 0.67–0.90, *p* = 0.001) for PD-L1 CPS ≥10 and PD-L1 CPS <10 subgroups, respectively. The HR for ICI/PD-L1 expression interaction was 0.80 (95% CI: 0.60–1.07, *p* = 0.129) (PD-1 inhibitor efficacy in PD-L1 CPS ≥10 patients *vs.* PD-L1 CPS <10 patients). The second most relevant interaction was ICI and region. Overall HR for PD-1 antibody *vs.* chemotherapy (reference) was 0.72 (95% CI: 0.63–0.83, *p* < 0.001) and 0.92 (95% CI: 0.68–1.25, *p* = 0.591) for Asia and extra-Asia subgroups, respectively. The HR for ICI/region interaction was 0.75 (95% CI: 0.50–1.12, *p* = 0.155) (PD-1 inhibitor efficacy in Asia patients *vs.* extra-Asia patients), but this was not statistically significant.

## Discussion

About 64% of patients in KEYNOTE-181 were squamous carcinoma. All the patients in ATTRACTION-3 and ESCORT were squamous carcinoma, so we only compared squamous carcinoma in these three studies. The three PD-1 inhibitors showed similar survival benefit in ESCC patients. In the subgroup analysis for survival, nivolumab seemed to be better than pembrolizumab in patients whose PS score is 1. This may be partially explained by the better safety profile of nivolumab. Based on the indirect analysis of AEs, camrelizumab had a significantly higher rate of all AE events than both pembrolizumab and nivolumab, and pembrolizumab had a numerically higher rate of all events than nivolumab. Similar results were found in ≥ grade 3 TRAEs. Therefore, the sequence of safety for these three PD-1 inhibitors was nivolumab followed by pembrolizumab and then camrelizumab. A recent systematic review and network meta-analysis for safety of ICIs in cancer also showed that nivolumab had the best safety profile (12). Camrelizumab had a high incidence rate of reactive cutaneous capillary endothelial proliferation (RCCEP) (13), which is 79% in the ESCORT study, but the incidence rate of grade 3 events was only 0.4% (9). Combining the safety and potential survival benefit, we recommend nivolumab for ESCC patients whose PS score is 1.

According to this indirect comparison, both pembrolizumab and camrelizumab had significantly higher ORR than nivolumab; moreover, camrelizumab had numerically higher ORR than pembrolizumab. Therefore, the sequence of ORR for these three PD-1 inhibitors was camrelizumab followed by pembrolizumab and then nivolumab. Moreover, camrelizumab had a significantly better PFS than nivolumab (*p* < 0.01), and camrelizumab has a tendency for longer PFS than pembrolizumab, and pembrolizumab also had better PFS than nivolumab, though these two differences were not significant, *p* = 0.06 and 0.29. In terms of PFS, camrelizumab is better than pembrolizumab followed by nivolumab. We would recommend camrelizumab or pembrolizumab for patients with better PS score and seeking for higher efficacy.

In the subgroup analyses, we found that PD-1 inhibitors were more effective in patients with PD-L1 CPS ≥10 than CPS <10 (HR interaction: 0.80), though it was not statistically significant. PD-L1 expression has been found as a predictor to PD-1 inhibitors in several diseases, such as gastric cancer (14, 15) and lung cancer (16, 17). However, the cutoff value for PD-L1 expression was controversial, and some studies used tumor-positive score (TPS) (16–18), while other studies use CPS (11, 12). In ESCC, PD-L1 CPS ≥10 is more commonly used (5, 7). Moreover, it seems that the result of pembrolizumab relies on the expression of PD-L1, while other PD-1 inhibitors, such as nivolumab and camrelizumab are independent of PD-L1 status. Based on our analysis, though the difference was not significant, we found that patients with PD-L1 CPS ≥10 benefit more from PD-1 inhibitors than those with CPS <10.

Subgroup analysis from clinical trials of PD-1 inhibitors has reported the impact of ethnic differences on outcomes (19, 20). In a meta-analysis to compare the therapeutic efficacy of PD-1/PD-L1 inhibitors in Asian and non-Asian patients, the author showed that Asian patients benefited significantly more than non-Asian patients in terms of OS (21). In our present analysis, we also found Asian ESCC patients benefit more from PD-1 antibody than non-Asia patients, though the difference was not significant. The exact mechanisms for different response to PD-1 inhibitors between Asian and extra-Asian populations are unclear, and potential explanations include difference in pharmacokinetic and genetic mutation profile (22, 23). Though both Japan and China belong to Asia, patients from different countries may benefit differently from PD-1 inhibitors (24). However, in the present study, we do not have enough data to compare the different efficacies between Japanese and Chinese patients.

The major limitation of this study was the indirect comparison analysis, which might compromise the evidence level. Secondly, expression of PD-L1 was measured with different antibodies, 28-8 pharmDx assay in ATTRACTION-3, 22C3 assay in KEYNOTE-181, and 6E8 assay in ESCORT, which might have influence on the evaluation of PD-L1expression. Thirdly, the chemotherapy used in the control group was not exactly the same among these three studies. In ATTRACTION-3, only paclitaxel and docetaxel were allowed, while in KEYNOTE-181 and ESCORT, except for paclitaxel, irinotecan was also one option. Paclitaxel and irinotecan have been confirmed to have no significant difference for OS in second-line treatment for advanced gastric cancer (25). Moreover, we only made the comparison among squamous carcinoma patients.

## Conclusions

Our study firstly compared nivolumab, pembrolizumab, and camrelizumab for advanced ESCC in second line and found the three PD-1 inhibitors provided comparable OS benefit. Noteworthy, nivolumab might further improve OS in patients whose PS score is 1. Based on the present analysis, the sequence for ORR or PFS of these three PD-1 inhibitors was camrelizumab followed by pembrolizumab and then nivolumab, while the sequence for safety was nivolumab followed by pembrolizumab and then camrelizumab. Therefore, we recommend nivolumab for ESCC patients with PS score of 1 and pembrolizumab or camrelizumab for patients with better PS and seeking for higher efficacy or longer PFS. Additional studies are warranted to confirm this finding.

## Data Availability Statement

The original contributions presented in the study are included in the article/supplementary material. Further inquiries can be directed to the corresponding authors.

## Author Contributions

Conception and design of this study were carried out by M-ZQ, Y-XZ, and BZ. Y-XZ, M-ZQ, PC, and Y-TS extracted the data and statistical analysis. All authors wrote the manuscript. All authors contributed to the article and approved the submitted version.

## Funding

This study was supported by the National Natural Science Foundation of China (NSFC: 82073377, 81903176, 81772587), Natural Science Foundation of Guangdong (2021A1515012439, 2019A1515011596), Science and Technology Program of Guangdong (2019B020227002); Guangdong Esophageal Cancer Institute Science and Technology Program (grant number M201809), CSCO-HengRui Oncology Research Fund (grant number Y-HR2018-184), the Third Outstanding Young Talents Training Plan, and Medical Scientist Program of Sun Yat-Sen University Cancer Center.

## Conflict of Interest

The authors declare that the research was conducted in the absence of any commercial or financial relationships that could be construed as a potential conflict of interest.

## Publisher’s Note

All claims expressed in this article are solely those of the authors and do not necessarily represent those of their affiliated organizations, or those of the publisher, the editors and the reviewers. Any product that may be evaluated in this article, or claim that may be made by its manufacturer, is not guaranteed or endorsed by the publisher.
